# A promising connection between BDNF and Alzheimer’s disease

**DOI:** 10.18632/aging.101518

**Published:** 2018-08-06

**Authors:** Maria Laura Giuffrida, Agata Copani, Enrico Rizzarelli

**Affiliations:** 1 Institute of Biostructures and Bioimaging, National Council of Research, IBB Catania, Catania, Italy; 2 Department of Drug Sciences, University of Catania, Catania, Italy; 3 Department of Chemical Sciences, University of Catania, Catania, Italy

**Keywords:** Aβ monomers, nerve growth factor, CREB, BDNF, copper and zinc

Our current understanding is that, in Alzheimer’s disease (AD), neurotoxic β-amyloid (Aβ) oligomers are formed from the self-association of Aβ monomers. Aβ monomers, which are naturally generated and secreted at firing synapses, are not mere bystanders of neuronal activity but, rather, regulators of neurotransmitter release [1] Monomers also appear needed for neuronal survival, given that the inhibition of endogenous Aβ production is lethal to cultured neurons. Hence, Aβ monomers rightfully fall into the scenario of factors, including the nerve growth factor (NGF) and the brain-derived neurotrophic factor (BDNF), able to regulate neurocognitive functions through the engagement of specific receptors and peculiar intracellular signaling(s). We previously showed that Aβ monomers have a broad neuroprotective activity mediated by the activation of the phosphatidyl-inositol-3-kinase pathway (PI3K/AKT), via the recruitment of type-1 insulin-like growth factor receptors (IGF-IRs) [2] Interestingly, as a downstream effect of PI3K/AKT activation, Aβ monomers are able to induce the activation of the cyclic AMP response element-binding protein (CREB), thus sustaining the transcription and release of the CREB-target gene, brain derived neurotrophic factor (BDNF) [3]. CREB-induced BDNF expression could be a converging point for different actors of synaptic maintenance. Accordingly, we have shown that NGF is able to induce BDNF expression *via* phosphorylation of CREB by a dual pathway comprising the extracellular signal-regulated kinase 1/2 and AKT kinase [4]. By enhancing NGF activity, copper and zinc, metal ions that take part in neurotransmission, are able to increase BDNF expression. The effect seems related to the presence of metal biding sites in the first 14 amino acid stretch at the N-terminus fragment of NGF. Incidentally, the NGF(1-14) sequence reproduces the signal transduction of the whole NGF peptide, thus activating CREB and promoting BDNF expression [4].

It is worth noting that BDNF is crucial for the maintenance of adult cortical neurons of the entorhinal cortex, whose early dysfunction contributes to the initial loss of short-term memory in AD [5]. The obvious suggestion is that a BDNF deficiency, resulting from a series of different factors, could shape the onset of AD neurodegeneration. These factors would include metal dyshomeostasis, lack of NGF support, loss of functional Aβ monomers, and appearance of toxic Aβ oligomers. Accordingly, as different from Aβ monomers, oligomers are not able to activate CREB and, therefore, they do not sustain BDNF transcription and release [3].

Within this scenario, as long as they last, Aβ monomers could restrain cognitive decline by maintaining not only BDNF levels [3], but also neuronal glucose homeostasis [6] In fact, by activating IGF-IRs and promoting the translocation of the Glut3 glucose transporter from the cytosol to the plasma membrane, Aβ monomers enhance glucose uptake in neurons under basal and depolarizing conditions [6].

So far, most of the treatment strategies for AD have been direct to lower total Aβ levels in the brain and have failed dramatically [7] The awareness that there is a pool of functional Aβ has led to on-going attempts (e.g., with aducanumab) to rebalance Aβ levels rather than reduce them strictly. We do not know yet whether Aβ monomer-sparing approaches will be sufficient to restore the dysfunctional CREB signaling that has been observed in mouse models of AD and even in patient cortices [6]. If so as with the conceivable use of the small Aβ and NGF fragments that mimic the full length protein [6,4], we would have found the way to overcome: i) the issues related to the use of BDNF as therapeutic agent, and ii) the potential serious side effects of a long-term pharmacological activation of the ubiquitous and oncogenic CREB protein.
